# Small size, big problems: insights and difficulties in prenatal diagnosis of fetal microcephaly

**DOI:** 10.3389/fnins.2024.1347506

**Published:** 2024-03-12

**Authors:** Leila Haddad, Efrat Hadi, Zvi Leibovitz, Dorit Lev, Yoseph Shalev, Liat Gindes, Tally Lerman-Sagie

**Affiliations:** ^1^Fetal Neurology Clinic, Wolfson Medical Center, Holon, Israel; ^2^Obstetrics & Gynecology Ultrasound Unit, Wolfson Medical Center, Holon, Israel; ^3^Faculty of Medicine, Tel Aviv University, Tel Aviv, Israel; ^4^Diagnostic Ultrasound Unit, The Institute of Obstetrical and Gynecological Imaging, Department of Obstetrics and Gynecology, Sheba Medical Center, Ramat Gan, Israel; ^5^Obstetrics & Gynecology Ultrasound Unit, Bnai Zion Medical Center, Haifa, Israel; ^6^Rappaport Faculty of Medicine, Technion-Israel Institute, Haifa, Israel; ^7^Medical Genetics Unit, Wolfson Medical Center, Holon, Israel; ^8^Pediatric Neurology Unit, Wolfson Medical Center, Holon, Israel

**Keywords:** fetal, microcephaly, ultrasound, diagnosis, neurosonography of fetus

## Abstract

Microcephaly is a sign, not a diagnosis. Its incidence varies widely due to the differences in the definition and the population being studied. It is strongly related to neurodevelopmental disorders. Differences in definitions and measurement techniques between fetuses and newborns pose a great challenge for the diagnosis and prognostication of fetal microcephaly. A false positive diagnosis can result (in countries where it is legal) in erroneous termination of pregnancy, where a false negative diagnosis might lead to the birth of a microcephalic newborn. Microcephaly in growth restricted fetuses deserves special attention and separate evaluation as it is an important prognostic factor, and not necessarily part of the general growth retardation. Several genetic syndromes incorporating microcephaly and intrauterine growth retardation (IUGR) are discussed. Deceleration of the head circumference (HC) growth rate even when the HC is still within normal limits might be the only clue for developing microcephaly and should be considered during fetal head growth follow up. Combining additional parameters such as a positive family history, associated anomalies, and new measurement parameters can improve prediction in about 50% of cases, and thus should be part of the prenatal workup. Advances in imaging modalities and in prenatal genetic investigation along with the emergence of new growth charts can also improve diagnostic accuracy. In this article, we review the different definitions and etiologies of fetal microcephaly, discuss difficulties in diagnosis, investigate the reasons for the low yield of prenatal diagnosis, and provide improvement suggestions. Finally, we suggest an updated algorithm that will aid in the diagnosis and management of fetal microcephaly.

## Introduction

Microcephaly is a descriptive term used to indicate a heterogeneous group of conditions that share a small head, and is regarded as a sign, not a diagnosis (Pilu et al., 1998; Dolk, 2008). Microencephaly is defined as an abnormally small brain and is used synonymously with microcephaly because a small head cannot harbor a normal sized brain (Yaniv et al., 2017). Its incidence varies widely due to differences in the definition and in the population being studied. It is strongly related to low intelligence quotient (IQ) scores, learning disorders, cerebral palsy, epilepsy, and other neurologic disorders (Ashwal et al., 2009).

Differences in definitions and measurement techniques between fetuses and newborns pose a great challenge for the diagnosis and prognostication of fetal microcephaly. During pregnancy, the physical examination and direct measurement of the head circumference (HC), are replaced by an ultrasound examination and measurement of the circumference of the bony ridge of the skull.

Prenatally, the head circumference is measured by ultrasound using calipers placed on the outer bony ridge of the calvarium, in the axial trans-thalamic plane and is not adjusted for gender (Chervenak et al., 1984). The definition of fetal microcephaly has far-reaching consequences and thus should be carefully discerned. Prenatal microcephaly is defined when the HC is more than 3 SD below the mean for gestational age (Chervenak et al., 1984, 1987). Jeanty’s reference range (Jeanty et al., 1984) is the most used despite being based on a small group of fetuses and despite the development of new reference ranges that in addition to being based on large populations, also utilize more advanced measurement modalities (Leibovitz et al., 2016b). The-3SD cut-off in the study by Chervenak et al., was found to be highly sensitive but not specific for the diagnosis of microcephaly, while using-4SD cut-off yielded higher specificity with no false positive cases (Chervenak et al., 1984, 1987; Malinger et al., 2003).

After birth, the head circumference is measured with a measuring tape wrapped around the widest possible perimeter of the head, surrounding the widest part of the forehead above the ears and the eyebrows and the most prominent occipital part of the head (Harris, 2015). Changes like molding or skin edema can under or over-estimate the real measurement. Microcephaly is defined when the HC is more than 2 SDs below the mean for age and gender. Severe microcephaly is defined as a HC of more than 3 SDs below the mean for age and gender (Leibovitz and Lerman-Sagie, 2018).

Assuming that the HC measurements are normally distributed, a HC below 2SD implies that 2.3% of fetuses will be diagnosed with microcephaly. This high rate is not in harmony with the actual prevalence of microcephaly at birth which is 0.54–0.56%. For a HC cut-off of 3SD below the mean, only 0.1% of children would be diagnosed with microcephaly, which corresponds to the published estimate of 0.14% of neonates (Ashwal et al., 2009; Leibovitz and Lerman-Sagie, 2018).

The prenatal diagnosis of microcephaly is challenging and prone to false positive and false negative results that can mislead pregnancy management and result (in countries where it is legal) in erroneous termination of pregnancy or the birth of a microcephalic infant.

Among school-age children the prevalence of microcephaly is 1.9%, rising to 15.4% among children with developmental disabilities. Around 80% of children with severe microcephaly (head circumference more than 3 SD below the mean for age and gender) have imaging abnormalities and severe developmental impairments (Ashwal et al., 2009). The incidence of intellectual disability and neurological abnormalities in the first year of life correlates with the number of SDs the HC is below the average: 11 and 51% for cut-offs of 2SD and 3SD, respectively (Dolk, 2008; Leibovitz and Lerman-Sagie, 2018).

In this article, we review the definitions and classifications of fetal microcephaly, discuss difficulties in diagnosis, investigate the reasons for the low yield of prenatal diagnosis, and provide improvement suggestions. Finally, we suggest an updated algorithm that will aid in the management of fetal microcephaly.

## Classifications of microcephaly

Microcephaly can be classified as congenital or postnatal.

Congenital microcephaly (CM) is divided into three categories: isolated microcephaly (also called pure microcephaly or microcephalia vera), syndromic microcephaly and non-syndromic microcephaly. In isolated microcephaly no other clinical cerebral or extra-cerebral abnormalities are detected including no developmental or intellectual disabilities. In syndromic microcephaly malformations of the cerebrum and/or extra-cerebral morphologic or functional anomalies are detected; and in non-syndromic microcephaly, neurological or psychiatric features are present, but without cerebral malformations or extra-cerebral abnormalities (Asif et al., 2023). Babies with congenital isolated microcephaly are typically born with a pathologically small HC (Leibovitz et al., 2022). Microcephaly primary hereditary (MCPH) refers to some of the genetic forms of CM.

Postnatal microcephaly is a term reserved for microcephaly that develops after birth. In these cases, the head circumference falls within the normal range at birth followed by the development of microcephaly over time due to slowing of brain growth (i.e Rett and Angelman syndrome), usually during the first 2 years of life. In some cases, the deceleration starts in the third trimester but at birth the HC is still in the normal range (i.e CASK related disorders) (Gafner et al., 2023). The development of postnatal microcephaly should be considered in fetuses displaying inadequate head growth (in at least 3 consecutive measurements) and deviation from the expected percentile.

Prenatal (congenital) microcephaly is characterized by a relatively static level of intellectual disability and brain volume deficit. In contrast, postnatal microcephaly is characterized by progressive brain degeneration (Wang et al., 2023).

## False positive diagnosis of fetal microcephaly

Not all fetuses who are diagnosed with microcephaly during the third trimester of pregnancy will become microcephalic newborns. In a study on 20 children between 2–6 years of age, who had fetal HC measurements between −2 and − 3 SD below the mean for gestational age during the third trimester, 18 (90%) were found to be normocephalic at birth, however three of them were microcephalic at the time of the neuropsychological examination. When these children were compared with children who had normal fetal cephalic biometry during the third trimester, and were matched for age and gender, there were no statistically significant differences between the two groups in cognition, language, and motor functioning. In contrast, there were statistically significant differences in behavioral problem scales (Stoler-Poria et al., 2010). The false positive rate is lower (about 40%) when defining fetal microcephaly as a HC of 3 or more SD below the mean for gestational age.

Another reason for the low accuracy in the prenatal diagnosis of microcephaly is the limited yield of the commonly used fetal head growth charts (Leibovitz and Lerman-Sagie, 2018). The most widely used growth charts for fetal head circumference are those of Chervenak. In his two studies on suspected microcephalic fetuses, Chervenak relied on Jeanty’s HC reference charts, which were developed in 1984 and based on a longitudinal assessment of 45 normal pregnancies. Chervenak’s studies included overall 40 suspected microcephalic fetuses, only 13 of which were proven to be microcephalic after birth (Chervenak et al., 1984, 1987). These references and cut-offs are still widely used despite their methodological limitations and the small size of the study groups.

In an attempt to improve biometric accuracy, sonographic estimations of fetal HC of 3,008 fetuses, performed within 3 days before delivery, were retrospectively reviewed and compared with actual measurements performed immediately at birth (Melamed et al., 2011). It was found that prenatal HC measurements significantly underestimated the actual postnatal measurements. This could be partially explained by the difficulty in obtaining the appropriate planes for measuring the fetal head during advanced pregnancy and possible molding of the fetal skull, making the HC measurement more difficult and less precise (Schwärzler et al., 2003; Leibovitz and Lerman-Sagie, 2018). Indeed, it was shown in this study that the gap between the measurements increased after 34 weeks and was found to be more pronounced in fetuses in a vertex presentation (Melamed et al., 2011). This high percentage of false positive diagnosis can lead to wrongful prenatal counseling or termination of pregnancies of normal fetuses in countries where it is allowed. This is accentuated by the absence of properly designed studies taking into consideration gender and ethnicity factors (Leibovitz and Lerman-Sagie, 2018). Methodological differences between prenatal and postnatal measurement of HC (discussed earlier in this paper), combined with different fetal HC measurement methods (calculated from biparietal diameter (BPD) and occipitofrontal diameter (OFD) as: HC = 1.62 × (BPD + OFD) or by measurement of an ellipse drawn around the outside of the calvarium) (Salomon et al., 2011), different refence charts, interobserver variability and differences in technical quality all contribute to diagnostic inaccuracies (Stoler-Poria et al., 2010; Leibovitz et al., 2016a).

## False negative diagnosis of fetal microcephaly

The prenatal diagnosis of microcephaly is also subject to false negative diagnosis. As previously discussed, the diagnostic criteria for microcephaly differ between fetuses and neonates, thus cases with a HC 2SD below the mean for gestational age, but not below 3SD, may go undiagnosed (Stoler-Poria et al., 2010). Furthermore, a progressive deceleration of head circumference growth during either the second or third trimesters may indicate the development of postnatal microcephaly, as observed in patients with Calcium/calmodulin-dependent serine protein kinase (CASK) loss of function variants causing microcephaly with pontocerebellar hypoplasia (MICPCH). MICPCH presents progressive microcephaly, intellectual disability, seizures, ophthalmological anomalies, and sensorineural hearing loss. Although microcephaly usually develops within the first months of life, a third of the female and half of the male patients already demonstrate microcephaly at birth (Gafner et al., 2023), with a head circumference below 2SD in around 50% of cases and show deceleration of head growth rate in the second and third trimester in about 50% of cases, but still prenatal diagnosis is rare (Gafner et al., 2023). This can be attributed partially to the development of these characteristics after the anatomic scan (performed at 22–24 weeks as currently recommended) (Salomon et al., 2022), and the lack of routine biometric follow up in the third trimester in some countries. This problem was also raised in a study on 7 fetuses with prenatally diagnosed microcephaly that was confirmed after birth. Only one fetus was diagnosed with microcephaly according to HC measurement before 22 weeks. The remaining 6 fetuses had normal head size measurements before 22 weeks of pregnancy and were diagnosed only after 27 weeks of gestation, emphasizing the importance of third trimester screening (Bromley and Benacerraf, 1995).

Gender differences in fetal HC can also contribute to the false negative diagnosis. The mean HC for male and female fetuses differs by 0.3–0.5 SDs, with males having larger heads, on average, than females. These differences may result in misdiagnosis of microcephaly (Schwärzler et al., 2003; Brawley et al., 2023).

Advances in genetic testing can reduce the false negative rate. Malinger et al. (2022) reported a case in which deceleration of HC growth rate was shown starting at 23 weeks of gestation, and at 37 weeks the HC was 2.4 SD below the mean for gestational age. The initial work-up was negative, MRI showed nonspecific brain findings, but the WES trio results revealed a heterozygous de-novo splicing (loss of function) variant in CCND2 gene. Mutations causing loss of function in this gene were reported for the first time in a case series only shortly before, by another group (Pirozzi et al., 2021), to be associated with microcephaly, growth restriction and neurodevelopmental disorders, thus enabling accurate prenatal diagnosis in this case.

## Microcephaly in SGA fetuses

Only a few studies have addressed microcephaly in small for gestational age (SGA) fetuses. Although it might be considered part of the general growth retardation, HC should be evaluated separately, as it is considered an important prognostic factor (Babson and Henderson, 1974; Gross et al., 1983). Microcephaly may be the cause of growth retardation due to reduced functioning of the hypothalamus or pituitary dysfunction (Dacou-Voutetakis et al., 1974; Villar et al., 1984). Intrauterine growth restriction has also been described in multiple disorders of pre-and post migrational microcephaly (Barkovich et al., 2012; Berkley and Abuhamad, 2012), and many teratogens are known to cause both microcephaly and growth retardation.

There is no consensus regarding the outcome of these children. Some reports have shown that low birth weight term infants with no additional diagnoses other than microcephaly, who were diagnosed by the age of 1 year, can expect a better outcome compared with those with a birth weight over 2,500 g and microcephaly (Brennan et al., 2008; Dolk, 2008). No significant difference was shown between children 2–6 years of age with symmetrical IUGR and children with a small head but normal *in-utero* body growth (Stoler-Poria et al., 2010), although there was a trend toward better motor functioning in the non-IUGR group.

**Figure 1:**
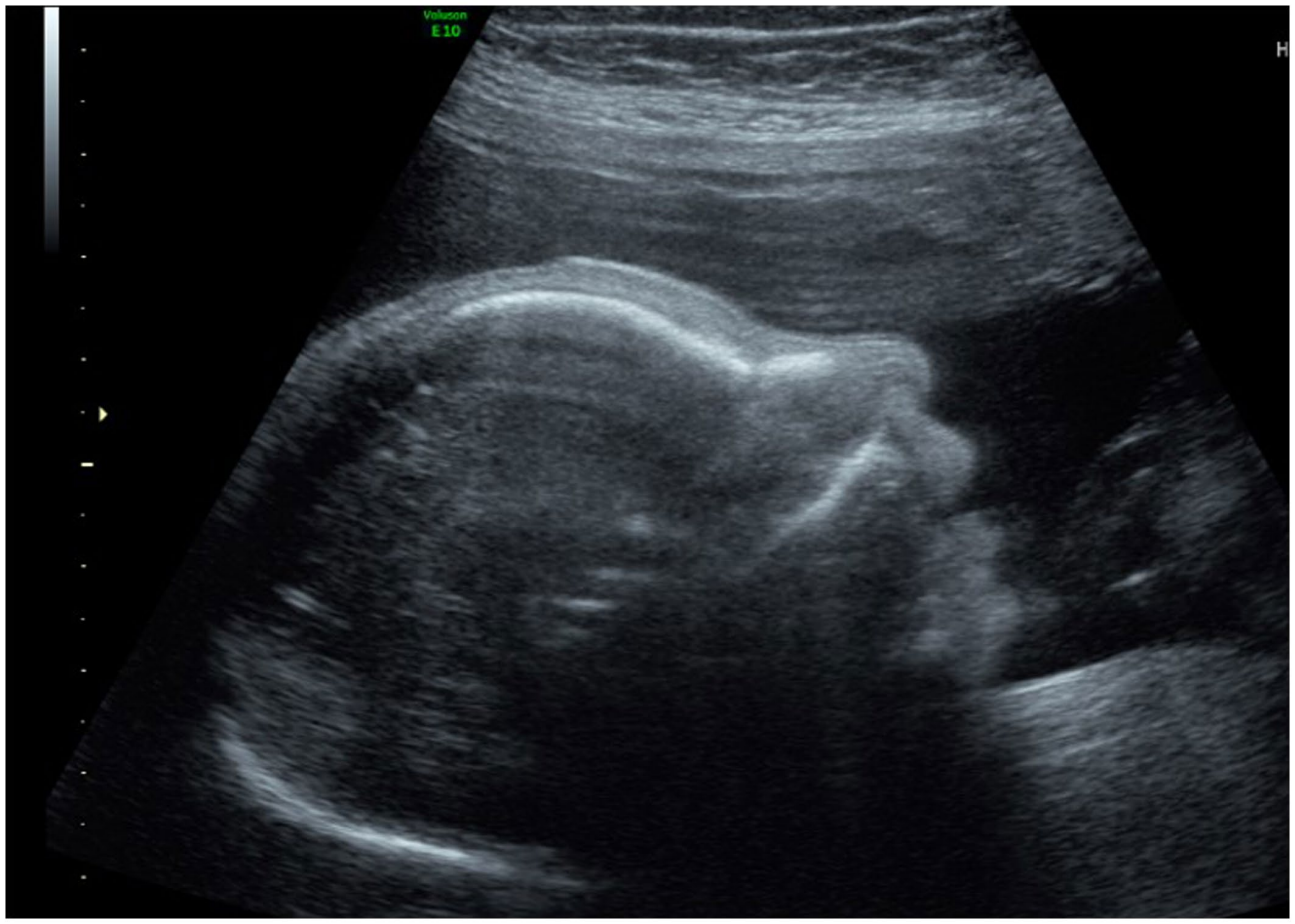
Sloping forehead.

Placental dysfunction leading to IUGR is an important risk factor for neurodevelopmental delay. The hemodynamic changes caused by placental dysfunction in late onset IUGR (after 32 weeks of gestation), cause reduced blood flow to the frontal lobe (Hernandez-Andrade et al., 2008; Cruz-Martinez et al., 2010; Baschat, 2011). The frontal lobe is the area of the brain most consistently affected in microcephaly (Pilu et al., 1998), and frontal love developmental delay has been reported to be indicative of microcephaly in fetuses (Goldstein et al., 1988). This often results in the high, sloping forehead (Figure 1) and enlarged subarachnoid space overlying the frontal lobes in the affected fetus (Persutte, 1998). The decrease in fetal head growth is a more important risk factor for suboptimal neurodevelopment than the overall degree of IUGR because it implies a serious compromise in placental function to the degree of eliminating the brain sparing effect.

Several genetic syndromes cause both microcephaly and IUGR. Microcephalic primordial dwarfism (MPD) is a group of disorders in which autosomal recessive microcephaly is accompanied by pre-and postnatal severe retardation of body growth. Many MCPH-and MPD-linked microcephaly genes encode centrosome proteins, and mutations in some centrosome genes can cause either MCPH or MPD. This raises the possibility of a common cellular origin for the associated brain development defects. Syndromes classified under MPD include Microcephalic Osteodysplastic Primordial Dwarfism (MOPD) types I and II, Seckel syndrome and Meier-Gorlin syndrome (Phan and Holland, 2021). The severe growth deficit is evident before birth, and features of skeletal dysplasia can also aid in prenatal diagnosis (Shalev et al., 2015). Below is a brief description of these syndromes:

**MOPD type II-Majewski syndrome**. Is the result of loss of function mutation in the pericentrin (PCNT) gene (Bober and Jackson, 2017). Growth restriction can be evident as early as the end of the first trimester. Other features of skeletal dysplasia can also be observed.

**MOPD type I Taybi-Linder syndrome**. A rare form of MOPD with severe microcephaly and severe symmetric growth restriction, which may be accompanied with brain malformations, skeletal dysplasia and dysmorphic facial features including beaked nose. It is caused by mutations in RNU4ATAC (Putoux et al., 2016).

**Seckel syndrome**. Is characterized by severe microcephaly with grossly normal brain architecture, intellectual disability, short stature, and bird-like appearance of the head. Growth restriction and microcephaly in Seckel syndrome can be diagnosed as early as the second trimester. CNS malformations that have been reported in prenatally diagnosed cases are agenesis of the corpus callosum, arachnoid cysts, cortical dysplasia, and encephaloceles. Other skeletal malformations can also be observed (Akkurt et al., 2019). Several gene variants have been identified as causes of Seckel syndrome, some of which are also known to be associated with MCPH. This suggests that the two conditions may be part of the same spectrum.

**Meier Gorlin syndrome**. Individuals with this rare syndrome have short stature, microtia and patellar aplasia or hypoplasia in addition to microcephaly. Micro-retrognathia and genu recurvatum can also exist, but there is variable expression of the phenotype. Intelligence can be normal. The syndrome is caused by a mutation in one of the genes of the pre-replication complex (ORC1, ORC4, ORC6, CDT1, and CDC6), which is essential for the initiation of DNA replication (Bongers et al., 2001; de Munnik et al., 2012).

**Congenital syndromic microcephaly**. Congenital syndromic microcephaly (associated with other systemic defects and dysmorphic features) is extremely genetically heterogeneous, including microdeletion or microduplication syndromes or single gene disorders (Pirozzi et al., 2018). These syndromes also manifest with IUGR. Some examples are chromosome abnormalities (Trisomy 21, Trisomy 13, Trisomy 18) and various contiguous gene deletion syndromes (4p deletion (Wolf-Hirschhorn) syndrome, 7q11.23 deletion (Williams) syndrome, 17p13.3 deletion (Miller-Dieker) syndrome). Other syndromes with multiple anomalies: Feingold, Cornelia de Lange, and Smith-Lemli-Opitz (Abuelo, 2007).

## How to improve detection and diagnostic accuracy

As the low prediction rate of prenatal microcephaly is partially due to relying on the old reference ranges that are based on studies with small sample sizes, using newer reference ranges based on large numbers and newer measurement techniques might optimize microcephaly prediction.

The applicability of the original 1984 Chervenak criteria for the diagnosis of fetal microcephaly, to a current population utilizing modern ultrasound equipment and techniques was examined in a retrospective study, including 27,697 ultrasound exams between 18-and 40-weeks of gestation. It was concluded that clinicians can either continue to use the 3SD cutoff suggested by Chervenak or develop a new dataset for different populations with statistical validation (Gelber et al., 2017).

In a retrospective study on 42 fetuses (Leibovitz et al., 2016a) previously diagnosed *in utero* as microcephalic using Jeanty’s reference range, with a 57% positive predictive value (PPV) in diagnosing microcephaly postnatally, (according to OFC measurement after birth or brain weight in autopsies after termination of pregnancy), two new references for fetal growth were used for evaluating the HC: the INTERGROWTH-21st project (Papageorghiou et al., 2014), and the new Israeli reference for fetal growth (Daniel-Spiegel et al., 2013). Both references were based on large populations and used modern measurement techniques (an electronic elliptical tool applied on the external skull border). The PPV of the INTERGROWTH and the Israeli population-specific reference were higher 61.5 and 66.7%, respectively relative to that of the conventional one (57.1%), but this did not reach statistical significance, probably due to the small number of cases studied. The authors found that adding a family history of microcephaly to the optimal HC cut-offs yielded a PPV of 100% using all three references (the conventional and the two new references).

Another cause of false positive diagnosis of microcephaly is an acrocephalic deformation due to craniosynostosis or molding of the fetal head. A new vertical cranial biometric measurement was used to address this entity: the foramen magnum-to-cranium distance (FCD). The FCD is measured between the foramen magnum and the upper inner cranial border along the posterior wall of the brainstem, in a precise mid-sagittal plane using a three-dimensional multiplanar display of a sagittal acquired sonographic volume of the fetal head (Leibovitz et al., 2016b). A normal reference range was developed based on measurements of 396 healthy fetuses between 15 and 40 weeks of pregnancy and an optimal FCD cut-off was defined and combined with HC to give maximal positive predictive value. This reference was retrospectively applied to 25 fetuses diagnosed with microcephaly with HC ≥ 3 SD below the mean for gestational age. The FCD was found to correlate well with gestational age and its combination with fetal HC measurement improved diagnostic accuracy (raised the PPV from 56 to 78%), and decreased the rate of false positive cases, without missing any of the cases of microcephaly at birth (Stoler-Poria et al., 2010; Leibovitz et al., 2016b).

Integrating other parameters like EFW <3rd% and fetal anomalies to the 3SD cutoff was found to increase the PPV from 57% (Leibovitz et al., 2016a) to 66 and 70%, respectively. Adding and family history to fetal HC 3SD below the mean for gestational age or setting the cutoff to 4SD below the mean, raised the PPV to 100% (Leibovitz and Lerman-Sagie, 2018).

Integrating sloping forehead and enlarged subarachnoid space overlying the frontal lobes (Goldstein et al., 1988) with HC measurements can also improve the PPV. Finally, gender specific charts can also improve diagnostic accuracy. As discussed earlier, the mean HC for male and female fetuses differs by 0.3–0.5 SDs, with males having larger heads, on average, than females (Brawley et al., 2023). These parameters and their contribution to the improvement of diagnostic accuracy must be further investigated in future studies.

## How to counsel parents

Counseling parents in cases of prenatally diagnosed microcephaly is difficult and challenging. Following the birth of one affected child and in the absence of any definitive factor, the estimated recurrence rate is 10% (Schwärzler et al., 2003).

In cases with associated US findings, abnormal genetic investigations or proven intrauterine infection, the prognosis is poor and abnormal neurodevelopment can be expected. In fetuses with isolated small HC, US and MRI examinations should be interpreted meticulously, to rule out gyration abnormalities. Cases with SGP or micro-lissencephaly have poor prognosis. These patterns usually develop late in pregnancy or sometimes after birth (Malinger et al., 2003).

There are no large studies reporting the correlation between fetal microcephaly and the severity of intellectual disability. Only one study that included 19 children who had fetal HC between 2 and 3 SD below the mean, showed they all had normal intelligence (Stoler-Poria et al., 2010).

A significant correlation between children’s HC at birth and maternal HC was found in several studies (Weaver and Christian, 1980; Hack et al., 1991; Stoler-Poria et al., 2010), It was suggested that a HC measurement outside the normal range should be adjusted to parental HC (Weaver and Christian, 1980). Having a parent with a small HC and normal intelligence was a positive predictive outcome factor (Hack et al., 1991).

## Evaluation of fetuses with suspected microcephaly

The prenatal investigation of suspected microcephaly should begin whenever the HC measurement is 2 SD or more below the mean for gestational age, or earlier when there is deceleration of HC growth rate. The evaluation should begin with confirmation of gestational age according to the earliest first trimester ultrasound. A detailed history should be obtained including maternal medical conditions (use of medications, alcohol consumption, substance abuse and possible exposure to other teratogens), family history of consanguinity, genetic disorders, or children with neurodevelopmental abnormalities. A thorough anatomical scan including fetal echocardiography and dedicated neuro-sonography can depict additional findings, suggesting syndromic microcephaly, which can affect the final diagnosis and prognosis. Intrauterine infection should be ruled out by testing antibodies for toxoplasma, rubella, CMV, herpes and Parvovirus. Zika should be tested in countries where it is endemic.

The HC of both parents and siblings should be measured. A family history of benign dominant microcephaly and normal intelligence usually carries a good prognosis.

Deceleration of fetal head circumference growth rate regardless of the reference range should be considered a “red flag” and warrants further evaluation (Gafner et al., 2023).

The importance of recording the HC growth rate has been demonstrated in a case report showing that the only prenatal sign of severe postnatal familial microcephaly was deceleration of head circumference growth at the end of gestation (Schwärzler et al., 2003).

**Figure 2:**
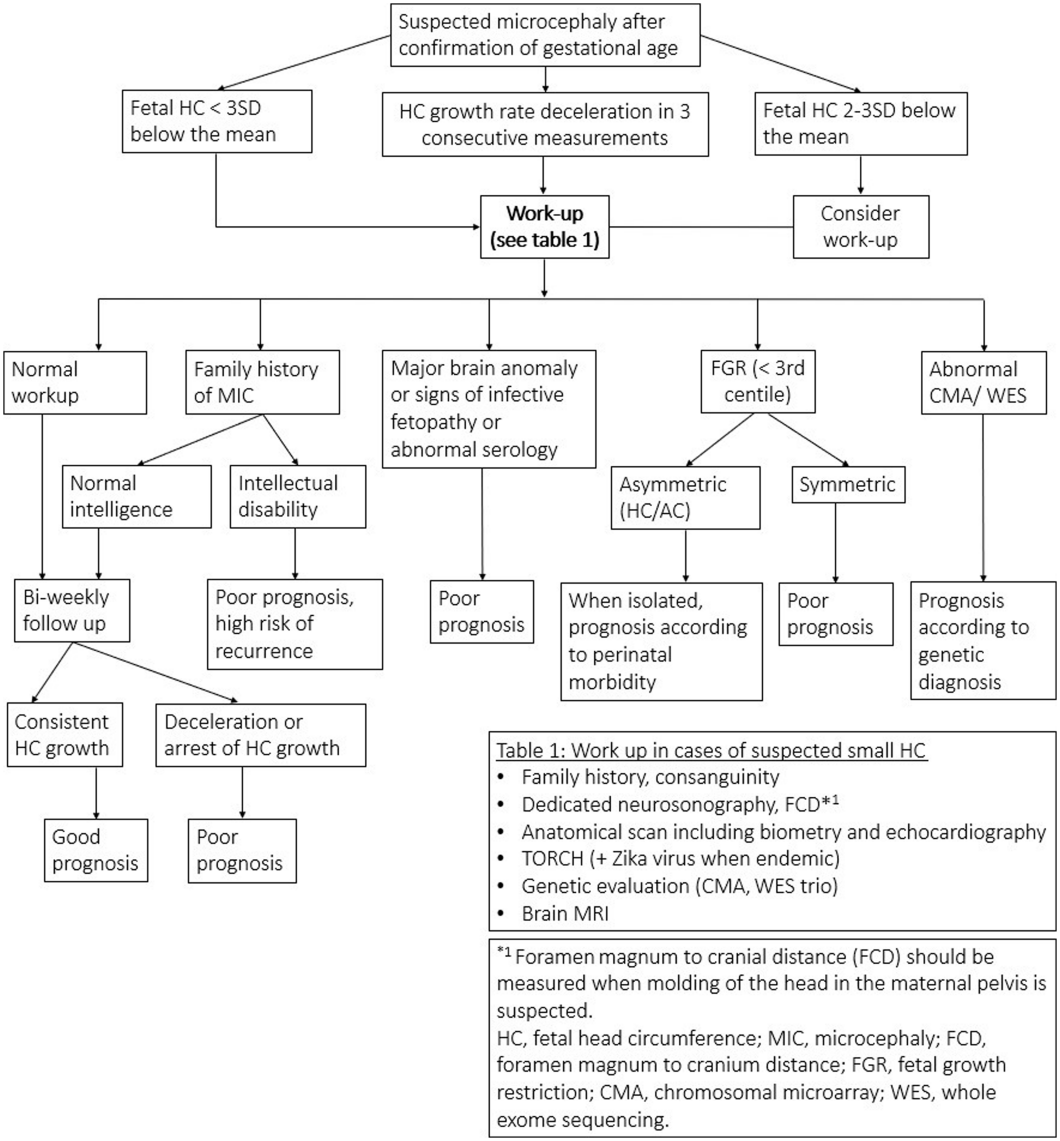
New work-up algorithm.

A new workup algorithm is suggested, based on the advances in both fields of ultrasound and genetic testing (Figure 2). The first workup algorithm was suggested by Malinger et al. (2003). Genetic testing was not included in this algorithm as little was known at that time. MRI was recommended only in cases with HC below 3SD, probably due to low availability.

In 2018, a revised algorithm was suggested by Leibovitz and Lerman Sagie, incorporating genetic investigations including CMA, while whole exome sequencing (WES) was reserved for cases with CNS/ extra CNS malformations, and cases with signs of infective fetopathy with negative infectious investigation. MRI was reserved for cases with deceleration of HC growth rate.

In our suggested algorithm, the new measurement parameter FCD is considered in the initial workup whenever molding of the fetal head in the maternal pelvis is suspected. Brain MRI and genetic testing are also incorporated in the initial workup.

In recent years, MRI has proved to be complementary to ultrasound and even superior in certain situations (ossification of the skull in advanced gestational age causing acoustic shadowing, molding of the fetal skull due to head position in the maternal pelvis and maternal obesity). It also has the advantage of measuring the brain parenchyma in addition to measuring the HC. Subtle findings indicating MCD may escape sonographic detection and can be depicted by MRI. The MRI is optimally performed at 32 weeks (Yaniv et al., 2017).

Genetic counseling and amniocentesis for chromosomal microarray (CMA) and whole exome sequencing (WES), should be offered whenever microcephaly is suspected. The application of CMA and WES in prenatal diagnosis, has led to a significant increase in diagnosis rate (Qi et al., 2020).

In the most recent study conducted in China (Wang et al., 2023), genetic causes were evaluated in 224 cases of prenatal microcephaly by CMA and WES. The positive diagnosis rate for WES (19.14%) was higher than both CMA and WES reported in other studies (Petrovski et al., 2019; Al-Kouatly et al., 2021; Sun et al., 2022). It was also found that the diagnosis rate by CMA was not significantly different between syndromic and primary microcephaly. More than half of the WES positive cases in this study (61.29%) were *de novo* variants, probably since trio testing was obtained. Among the 31 pathogenic variants identified by WES, 29.03% were autosomal dominant and 41.94% were autosomal recessive. This finding matched the genetics of microcephaly described in previous studies (Rump et al., 2016).

## Conclusion

Prenatal prediction of true congenital microcephaly and the clinical consequences remains difficult. Different pre and postnatal measurement techniques, old reference ranges based on small numbers and outdated equipment and different etiologies and mechanisms of microcephaly place great challenges when counseling the future parents.

Prenatal diagnosis of microcephaly by serial sonographic measurements of fetal head circumference has been recommended but should be regarded with caution as the head circumference measurements do not fall appreciably below normal centiles until the third trimester of pregnancy. Special attention should be taken in cases of deceleration of the growth rate even when the HC is still within normal limits (between 2–3 SD below the mean for gestational age). This deceleration may be the only clue for developing microcephaly.

Recent studies have concluded that combining a positive family history, associated anomalies, SGA, and stricter HC cut-offs can improve prediction in about 50% of cases. The use of the new vertical cranial measurement should be part of the prenatal workup any time a vertical deformation/ acrocephalic shape of the head is suspected.

MRI and genetic testing can improve the detection rate of microcephaly and therefore are recommended as part of the initial workup.

## Author contributions

LH: Investigation, Writing – original draft. EH: Writing – original draft. ZL: Supervision, Writing – review & editing, Conceptualization. DL: Writing – review & editing. YS: Writing – review & editing. LG: Writing – review & editing, Supervision. TL-S: Conceptualization, Supervision, Writing – review & editing.
